# Seizures of illicit substances for personal use in two Italian provinces: analysis of trends by type and purity from 2008 to 2017

**DOI:** 10.1186/s13011-019-0229-y

**Published:** 2019-09-18

**Authors:** Patrizia Verri, Cecilia Rustichelli, Anna Ferrari, Filippo Marchesi, Carlo Baraldi, Manuela Licata, Daniele Vandelli, Federica Palazzoli, Francesco Potì, Enrico Silingardi

**Affiliations:** 1 Forensic Toxicology Laboratory; Department of Biomedical, Metabolic and Neural Sciences, University of Modena and Reggio Emilia, Via del Pozzo, 71, 41124 Modena, Italy; 2 Department of Life Sciences, University of Modena and Reggio Emilia, via G. Campi, 103, 41125 Modena, Italy; 3 Unit of Medical Toxicology, Headache Centre and Drug Abuse; Department of Biomedical, Metabolic and Neural Sciences, University of Modena and Reggio Emilia, Via del Pozzo, 71, 41124 Modena, Italy; 4 School of Medical Toxicology, University of Modena and Reggio Emilia, Via del Pozzo, 71, 41124 Modena, Italy; 5 Unit of Neuroscience, Department of Medicine and Surgery, University of Parma, Via Volturno, 39F, 43125 Parma, Italy

**Keywords:** Drug seizure, Illicit substance, Cannabis, Cocaine, Heroin, New psychoactive substance, Synthetic cathinone, Phenylethylamine

## Abstract

**Background:**

The use of illicit substances represents one of the most difficult problems to confront in the health system. Drug use is a global problem but is not uniform throughout the world, within the same country and changes over time. Therefore, knowing the illicit substances that are used in a territory is essential to better organize health services in that specific geographical area. To this aim, we analysed 4200 samples confiscated from individuals who held them for personal use by police forces in the Italian provinces of Modena and Reggio Emilia from 2008 to 2017.

**Methods:**

The suspected samples were screened by gas-chromatography-mass spectrometry (GC-MS) and by liquid chromatography-tandem mass spectrometry (LC-MS/MS); all samples were subsequently analysed by gas chromatography-flame ionization detector (GC-FID) for quantitative analyses.

**Results:**

Cannabis was the most seized illicit substance (70.7%). Over the study period, the number of seizures of herb with a high content of Δ^9^-THC increased. The number of cocaine seizures remained stable (total 16.1%), but the median purity of seized cocaine increased to 75% in 2017. Heroin seizures decreased over time, but the median purity of seized heroin reached 16.8% in 2017. In almost all the years, heroin samples with a purity exceeding the 97.5 percentile were found. Especially from 2014, the range of seized substances increased and started to include synthetic cathinones, phenylethylamines, UR-144, LSD, psilocybe, prescription opioid and hypnotics. In two cases, tramadol together with tropicamide was seized. Most of the seizures involved male subjects and 82% of the seizures were from individuals younger than 35 years of age.

**Conclusions:**

The persistence of old illicit drugs and the rapid emergence of new psychoactive substances represented a serious challenge for public health in the studied Italian area. Some useful interventions might be: informing mainly young people about the possible complications of cannabis use; implementing standardized procedures to diagnose and treat cocaine-related emergencies in hospitals; increasing the distribution of naloxone to antagonize possible heroin overdoses; equipping laboratories to be able to identify the new psychoactive substances.

## Background

The use of illicit drugs is one of the major causes of health care costs worldwide and, at the same time, represents one of the most difficult problems to deal with in the health system [1]. Drug use affects a huge number of people; approximately 31 million people have had a disorder related to the consumption of illicit substances [2]. As people who use drugs have a higher rate of medical [1] and psychiatric [3] comorbidities than the general population, they consequently use more health services such as first aid [4], hospital admissions and re-admissions after the first hospitalization [5, 6]. Moreover, the pathologies of people who use drugs vary according to the used substances [1]. People who use cocaine and amphetamine suffer from cardiovascular events more than the general population [7, 8], while lung diseases are more common in crack smokers [9], half of the nearly 16 million people who use drugs by injection are HCV-positive, and approximately 18% are HIV-positive [10].

In addition, the use of illicit substances varies over time, is not uniform throughout the world, within the same country or between large and small cities. Among the general population in Europe, cannabis is the most frequently used substance (5.2%), followed by cocaine (0.74%) and opioids (0.57%), but in Eastern Europe, opioids are in the second place (0.85%) and cocaine in the third (0.27%) [11]. In Italy, wastewater analysis in 2010–2014 showed that cannabis and cocaine were used significantly more in the centre of the country, and low cocaine consumption was typical of small and medium cities [12]. Within the same geographical area, even the range of substance purity can be very wide. In an Italian study on the seizures carried out by the police forces in the 2013–2016 period, the mean purity of heroin samples averaged 30% in northern and 13% in southern regions [13].

The large seizures by the police forces provide useful information that, however, cannot be used as direct indicators of the illicit substances present in the local market. In fact, the operations of the police forces also intercept the batches of illicit drugs, usually with a high degree of purity, which are in transit in the territory and are destined for other regions or countries. Moreover, they are hardly able to highlight new psychoactive substances (NPS) as individual person usually purchases them through online stores or on the darknet [14] and therefore, NPS are likely to evade detection and seizure by police. The NPS represent a growing and worrying phenomenon due to their little-known toxicity; moreover, their large structural diversity and the lack of knowledge of the pharmacokinetics, and in particular metabolism, make their use hard to quickly identify and, in clinical settings, various NPS could not be detected by the available routine tests [15].

In Italy, the analyses of seizures for suspected illicit products are mandatory both in the cases of dealing and trafficking (Art. 73, criminal offence punishable by imprisonment) and in the case of possession of illicit drugs for personal use (Art. 75, administrative offence, punishable by administrative sanctions), in conformity with D.P.R. 309/90 and subsequent amendments (Italian law regulating illicit substances). Therefore, the analysis of the seizures exclusively confiscated to individuals for personal use could provide a more accurate picture of the type and characteristics of the illicit substances, including NPS, which are used within a community. This information would be of great importance to better organize the healthcare services in the context of a specific territory.

The aim of our study was to analyse all seizures suspected of illicit substances that were confiscated by police forces in conformity with Art. 75 D.P.R.309/90 (possession for personal use) in the provinces of Modena and Reggio Emilia (Italian provinces that were hit by a strong earthquake in 2012 that caused 27 victims), from 2008 to 2017. Moreover, we compared the results obtained throughout the examined 10–year period by considering the type and purity of the found substances and the age and sex of the subjects from whom the substances were seized. We hope that the information deriving from our study can be translated into interventions aimed at addressing at least some of the health and social problems of the individuals who use substances and of the community in which they live.

## Methods

### Study area

**Fig. 1:**
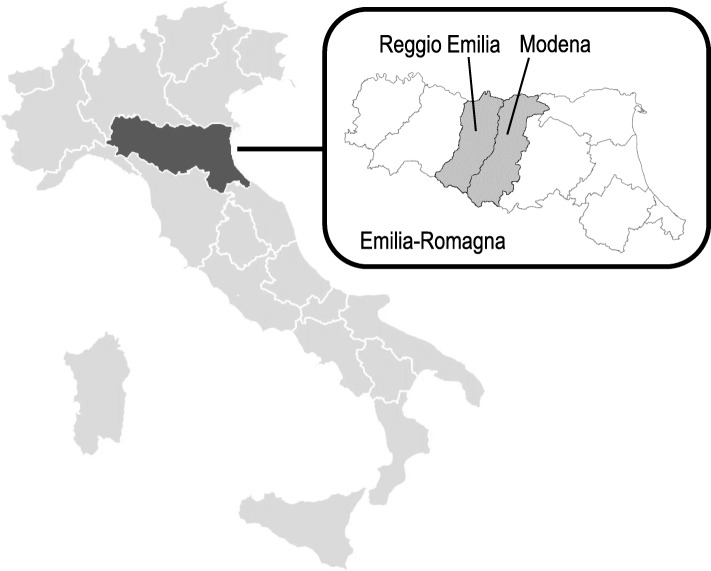
Layout of the studied area (provinces of Modena and Reggio Emilia) within the Emilia-Romagna region

The provinces of Modena and Reggio Emilia are located in the centre of the Emilia-Romagna region (Fig. 1), in an Italian geographical area characterized by an extensive national and international transport network and with a population exceeding 1,200,000 inhabitants. This area represents one of the major European economic communities with important industries in various fields, including food, engineering, ceramic, textiles, chemistry and biomedical science [16].

### Seized samples

The illicit drug samples were confiscated from subjects stopped while violating Art. 75, D.P.R. 309/90 by Italian police forces (Carabinieri, State police, Municipal police and Financial police) operating in the provinces of Modena and Reggio Emilia over 10 years, from 2008 to 2017. The seized samples (herbal material such as buds and leaves, resin material such as small pieces, slabs/blocks, handmade cigarettes, powder, tablets, crystals, liquids, blotter and mushrooms) were analysed in the Forensic Toxicology Laboratory of the University of Modena and Reggio Emilia (Italy). Screening analyses to identify the presence of psychoactive substances in the suspected samples were performed by gas-chromatography-mass spectrometry (GC-MS) and by liquid chromatography-tandem mass spectrometry (LC-MS/MS) procedures; conversely, the subsequent quantitative determinations of the illicit analytes in the same samples were carried out by gas chromatography-flame ionization detector (GC-FID).

All procedures were conducted in compliance with the legal regulations and the chain of custody. The laboratory analyses were carried out on non-biological samples, at the request of the Judicial Authority in the context of administrative proceedings. The samples to be analysed were delivered to the laboratory already coded with a protocol number. Gender and age, if indicated, were linked to the code. The identity of the subjects and the results of the analyses could only be linked by the Judicial Authority. The Forensic Toxicology Laboratory had no possibility of tracing or identifying the subject to whom the suspected illicit substance had been seized. The institutional review board of the University of Modena and Reggio Emilia assessed this study as not requiring ethical approval.

Experimental details concerning chemicals, sample processing procedures, analyses by GC-MS, LC-MS/MS [17–20] and GC-FID and method validation [21, 22] are reported in the Additional file 1.

### Data and statistical analysis

All data (age and sex of the subjects halted for personal possession of illicit drugs, year of the seizure, qualitative analytical results, i.e., substance type, and quantitative analytical results, i.e., purity of the seized samples) were added to a database and analysed with STATA IC13 software. A complete descriptive analysis of the data was carried out. The annual number of seizures for each illicit substance throughout the 10–year period (2008–2017) was compared by Fisher’s exact test. The trends in the odds of seizures from 2008 to 2017 were calculated for each substance by logistic regression analysis using the binary variable indicating the presence/absence of the substance in the seizures of each year (probability of event/non-event) as the dependent variable and the year as the independent variable. The proportions per year of each substance were compared throughout the 10–year period using the Chi-square test of homogeneity of odds and the Chi-square score test for trend of odds. The annual contents of each illicit substance in the seizures were evaluated in terms of median value and 25th–75th percentiles. Moreover, we considered the number of seizures from different age classes and the possession of more than one illicit drug (number of subjects and substance type). Finally, the mean subject age for each substance type and year and the seizure trend based on gender and substance type throughout the studied period were compared using one-way analysis of variance, and the Chi-square test of homogeneity of odds and the Chi-square score test for trend of odds. Continuous variables were expressed as the mean ± standard deviation (SD), the categorical variables as proportions and percentages. *P* < 0.05 was chosen to indicate significance for all the tests.

## Results

Overall, from 2008 to 2017 (Table 1), the police forces sent 4200 samples suspected to contain illicit substances to the Forensic Toxicology Laboratory. The number of seizures in 2008 was significantly lower than all other years (*P* < 0.0001, Fisher’s exact test). In 2013, the number of seizures significantly increased compared to all previous years (*P* < 0.01); however, the highest value was in 2014, which was significantly higher than all other years, except 2015 (*P* < 0.05, Fisher’s exact test). In each considered year, the most seized substance was cannabis (resin and herb). Specifically, cannabis derivatives accounted for 80% of all seizures in 2013, 2016 and 2017. In 2013, 2014 and 2017, the seizures of cannabis herb were more common than those of cannabis resin. Cocaine was always the second most seized substance after cannabis, with a slight decline in the seizure frequency throughout the study period. The minimum percentage of cocaine seizures occurred in 2016, and the maximum percentage in 2008. Heroine ranked third; the lowest percentage of heroine seizures occurred in 2017, and the maximum in 2009. Other substances accounted for a limited number of seizures in all the studied years.

**Table 1:** Seizures in the provinces of Modena and Reggio Emilia per year and by detected substance

		Cannabis					
Year	Samples	Resin	Herb	Total	Cocaine	Heroin	Other drugs
	n (%)	n (%)	n (%)	n (%)	n (%)	n (%)	n (%)
2008	252 (100)^a^	137 (54.4)	16 (6.3)	153 (60.7)	62 (24.6)	36 (14.3)	1 (0.4)
2009	368 (100)^b^	171 (46.5)	38 (10.3)	209 (56.8)	80 (21.7)	77 (20.9)	2 (0.5)
2010	351 (100)^c^	145 (41.3)	66 (18.8)	211 (60.1)	65 (18.5)	62 (17.7)	13 (3.7)
2011	322 (100)^c^	137 (42.6)	60 (18.6)	197 (61.2)	60 (18.6)	55 (17.1)	10 (3.1)
2012	354 (100)^c^	171 (48.3)	87 (24.6)	258 (72.9)	57 (16.1)	33 (9.3)	6 (1.7)
2013	512 (100)^d^	192 (37.5)	225 (43.9)	417 (81.4)	59 (11.5)	34 (6.6)	2 (0.4)
2014	575 (100)^e^	144 (25.0)	252 (43.8)	396 (68.9)	87 (15.1)	79 (13.7)	13 (2.3)
2015	532 (100)^f^	242 (45.5)	142 (26.7)	384 (72.2)	89 (16.7)	49 (9.2)	10 (1.9)
2016	495 (100)^g^	232 (46.9)	165 (33.3)	397 (80.2)	54 (10.9)	37 (7.5)	7 (1.4)
2017	439 (100)^h^	136 (31.0)	212 (48.3)	348 (80.0)	63 (14.5)	20 (4.6)	8 (0.9)
Total	4200 (100)	1707 (40.6)	1263 (30.1)	2970 (70.7)	676 (16.1)	482 (11.5)	72 (1.7)

Number of seizures in

^a^ 2008; lower than all other years (*P* < 0.0001);

^b^ 2009; lower than years 2013–2017 (*P* < 0.05);

^c^ 2010, 2011 and 2012; lower than years 2013–2017 (*P* < 0.005);

^d^ 2013; higher than all previous years and 2017 (*P* < 0.01), lower than 2014 (*P* < 0.05);

^e^ 2014; higher than all other years (*P* < 0.05), except vs 2015;

^f^ 2015; higher than 2008–2012 (*P* < 0.0001) and 2017 (*P* < 0.005);

^g^ 2016; higher than 2008–2012 (*P* < 0.0001) and 2017 (*P* < 0.05), lower than 2014 (*P* < 0.01);

^h^ 2017; higher than 2008–2012 (*P* < 0.05); Fisher’s exact test

Based on the binary logistic regression models performed, from 2008 to 2017 seizures of cannabis resin had an average percentage decrease per year of 5.5% (3.2–7.7%), cannabis herb had an increase of 20.1% (17.4–22.8%), total cannabis derivatives had an increase of 12.6% (10.1–15.1%), cocaine had a decrease of 7.9% (4.9–10.9%) and heroin had a marked decrease of 14.3% (10.8–17.8%) (*P* < 0.001 for all substances).

**Fig. 2:**
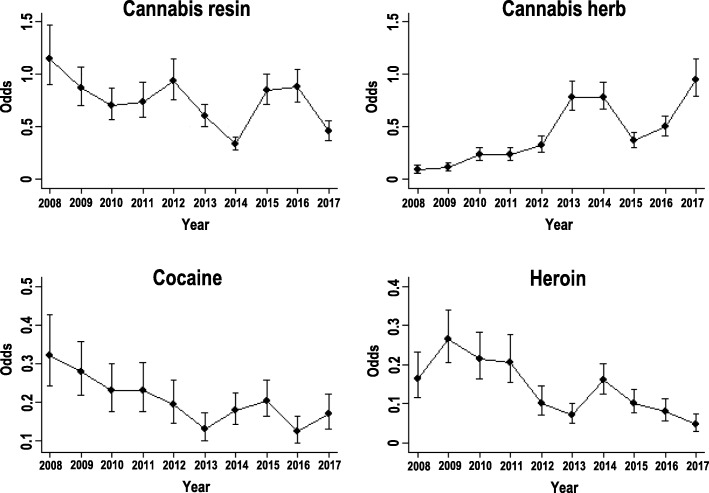
Odds of seizures per year and by detected substance; Chi-square test for homogeneity of odds. Cannabis resin: the odds in 2008 were higher than in all other years, except 2009, 2012 and 2016; the lowest values were in 2014 and 2017; the odds in 2013 were lower compared to 2009, 2012, 2015 and 2016 (*P* < 0.05); Cannabis herb: minimum values were in 2008 and 2009; the odds in the three-year period 2010–2012 were lower than in all years from 2013 onwards, except 2012 vs. 2015; the maximum values were in 2013, 2014 and 2017; the odds in 2016 were higher than in 2012 and 2015 (*P* < 0.05); Cocaine: the odds in 2008 were higher than in 2012–2017; in 2009 were higher than in 2013–2017, except 2015; the odds in 2010–2011 and in 2014 were higher compared to 2016; the odds in 2013 were lower than in 2008–2011 and 2015; the odds in 2016 were lower than in other years (*P* < 0.05), except 2013 and 2017; Heroin: the odds in 2008 were higher compared to those in the years 2013 and 2015–2017; in 2009 were higher than 2008 and 2012–2017; the values in 2010–2011 were higher than in 2012–2017, except 2014; the odds in 2013, 2015 and 2016 were lower compared to 2008–2011 and 2014; the odds in 2017 were lower than in all other years (*P* < 0.05), except 2013 e 2016

The odds ratio of the seizures for each illicit substance (Fig. 2) highlighted peculiar changes. The proportion of cannabis resin seizures increased in 2012, 2015 and 2016, while the odds ratio values in 2014 and 2017 were significantly lower than those in all the other years (*P* < 0.05, Chi-square test). For cannabis herb, the odds ratio in 2017 was significantly higher than the one of all the other years (*P* < 0.05, Chi-square test), except 2013 and 2014. For cocaine, the odds ratio of seizures decreased slightly until 2013 and then it remained quite stable. The odds ratio value for heroin increased considerably in 2009, then the values decreased markedly until 2013, when they remained quite stable.

The other seized substances (different from cannabis, cocaine and heroin) (Table 2) represented a small percentage, less than 2%. However, they accounted for various compounds, such as synthetic cathinones (mephedrone, butylone, 4-MEC, 3-MMC, pentedrone), old (amphetamine, MDMA) and new phenylethylamines (2C-B), a synthetic cannabinoid (UR-144), hallucinogens (ketamine, LSD, psilocybe), prescription opioids (methadone, buprenorphine, tramadol) and hypnotics (lormetazepam, zolpidem). Mephedrone was already seized in 2010 and recurred throughout the last years. Especially from 2014, the seizures included substances that were never previously present.

**Table 2:** Samples containing other substances different from cannabis, cocaine and heroin, per year and by substance

Substance (form)	Samples n (%)	2008	2009	2010	2011	2012	2013	2014	2015	2016	2017
Ketamine (powder)	7 (9.7)	1		2	1				1	2	
Methadone (liquid)	17 (23.6)		2	6	5			1	1	1	1
Buprenorphine (tablet)	1 (1.4)				1						
Amphetamine (tablets, powder)	10 (13.9)			1	1	3	1	3		1	
Methamphetamine (crystals)	9 (12.5)						1	2	1	2	3
MDMA^a^ (tablets, powder)	13 (18.1)			3	2	3		5			
Mephedrone (tablets)	3 (4.2)			1					1		1
UR-144 (resin material)	1 (1.4)							1			
LSD^b^ (blotter)	1 (1.4)								1		
2C-B^c^ (blotter)	1 (1.4)							1			
Psilocybe (mushrooms)	1 (1.4)								1		
Butylone (tablet)	1 (1.4)								1		
4-MEC ^d^ (tablet)	1 (1.4)								1		
3-MMC ^e^ (tablet)	1 (1.4)										1
Pentedrone (tablet)	1 (1.4)										1
Tramadol ^f^ (tablets)	2 (2.8)								1		1
Hypnotics (tablets): Lormetazepam Zolpidem	2 (2.8)								1	1	
Total (%)	72 (100)	1 (1.4)	2 (2.8)	13 (18.0)	10 (13.9)	6 (8.3)	2 (2.8)	13 (18.0)	10 (13.9)	7 (9.7)	8 (11.1)

^a^ MDMA: 3,4-methylene-dioxymethamphetamine; ^b^ LSD: D-lysergic acid diethylamide; ^c^ 2C-B: 4-bromo-2,5-dimethoxybenzeneethanamine; ^d^ 4-MEC: 4-methylethcathinone; ^e^ 3-MMC: 3-methylmethcathinone; ^f^ tropicamide (eye drops) was seized simultaneously

No seized material presented the simultaneous presence of more than one illicit substance; however, a minority of subjects (*n* = 164/4032, 4%) possessed more than one material, mostly heroin and cocaine (*n* = 160/164, 97.6%). In two cases, tramadol and tropicamide were seized together. The number of people possessing multiple substances, although small, tripled from 2008 to 2017.

**Fig. 3:**
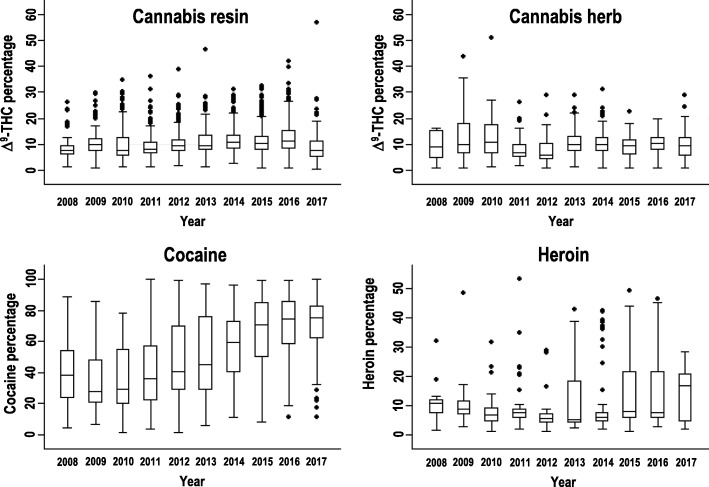
Box plot of potency (Δ^9^-THC content) and purity (cocaine, heroin) by year. Median, horizontal line; boxes, 25th–75th percentiles; whiskers, 2.5th–97.5th percentiles; dots, outliers

The median Δ^9^-tetrahydrocannabinol content (Δ^9^-THC, Fig. 3) in samples of cannabis resin did not significantly change throughout the study period and averaged 9.5%. The highest potency value (nearly 60%) was found in a sample seized in 2017, and several samples exhibited contents exceeding the 97.5th percentile. The median Δ^9^-THC content did not significantly change over the study period for cannabis herb, too; the average level found for cannabis resin was 9.5%. The potency of cannabis herb remained quite constant over time; indeed, only a few samples exhibited contents exceeding the 97.5th percentile. The median purity of the seized cocaine increased throughout the study period, especially since the beginning of 2014, and reached a percentage value of 75% in the last 2 years (74.6 and 75.5%, respectively). The samples outside the 2.5th–97.5th percentiles in 2016 and 2017 were outliers with low purity values. For heroin seizures, the median purity ranged from 5 to 10% in 2008 until 2016, but in 2017, it reached a value of 16.8%. Samples with purity exceeding the 97.5th percentile were found in most of the examined years; these samples had a heroin content more than twice the corresponding median levels.

The age of 3088 of the 4032 subjects (76.6%) stopped while violating Art. 75, D.P.R. 309/90 (possession of illicit drugs for personal use) was known. The mean age was 27 ± 8.7 years (range: 14–77 years). In general, the majority of seizures (82%) were confiscated from subjects younger than 35 years. Approximately half of the stopped individuals (*n* = 1523, 49.3%) were between 14 and 24 years old, 32.7% of the individuals were between 25 and 34 years old (*n* = 1009), 13.1% (*n* = 405) of the subjects were in the 35–44 age group and 4.2% of the subjects were between 45 and 54 years old (*n* = 130); only 21 subjects (0.7%) were in the oldest age group (55–77 years old).

**Fig. 4:**
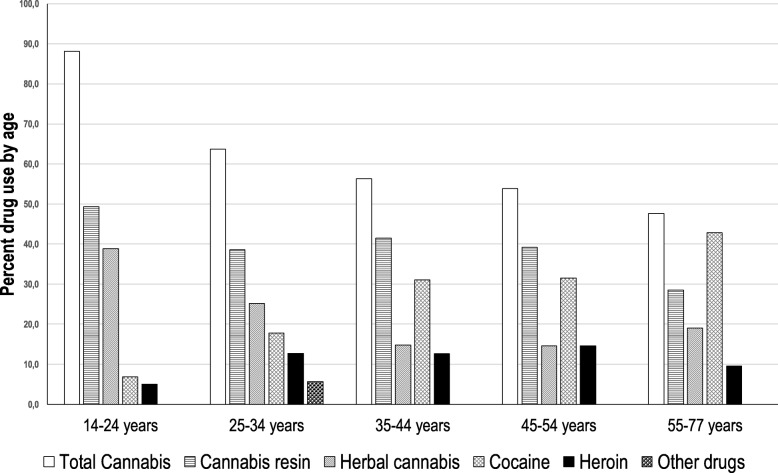
Percentage of seized substances by age class of the subjects. 14–24 years (*n* = 1523), 25–34 years (*n* = 1009), 35–44 years (*n* = 405), 45–54 years (*n* = 130) and 55–77 years (*n* = 21)

In all age groups, cannabis was the most seized substance (Fig. 4); the seizures of cannabis resin were always prevalent, except in the 55–77 age group. Among the very young people, those who were 14 to 24 years old, nearly 90% of the seizures were cannabis (resin, approximately 50%; herb, approximately 40%). In the 25–34 age group, cocaine seizures were appreciably increased and heroin seizures were increased to a lesser extent, while cannabis seizures decreased. Cocaine possession was increased among people older than 35 years; in terms of seizure frequency, cocaine was the second most common substance seized from the 35–54 age group and the most common substance seized from the over-55 age group. Heroin was always the least seized substance in all age groups, except in the 45–54 age group, where the percentage of heroin seizures was equal to the one of cannabis herb seizures (14.6%). Other substances, including NPS, had been confiscated only to subjects aged between 25 and 34 years.

Subjects possessing cannabis resin (mean age: 26.3 ± 8.4 years) or cannabis herb (mean age: 24.3 ± 7.1 years) were significantly younger than those possessing cocaine (mean age: 32.6 ± 9.4 years) or heroin (mean age: 30.2 ± 8.26 years) (*P* < 0.0001). Subjects who had been confiscated other substances, including NPS, had an average age of 29.4 ± 6.8 years and were significantly younger than those who held cocaine and heroin and older than those who held cannabis resin (*P* < 0.05, one-way analysis of variance). No significant differences were found throughout the examined period among the mean subject age for each substance.

Most seizures involved males (*n* = 3996/4200, 95.1%), without significant differences throughout the years (*P* = 0.53, Chi-square test for homogeneity of odds). Females (Table 3) were a small minority among a large majority of men, without significant differences by type of illicit substances (*P* = 0.2106, Chi-square test for homogeneity of odds).

**Table 3:** Seizure number per gender and by detected substance

Substance	Seizure number	Females n (%)	Males n (%)
Cannabis resin	1707	80 (4.7)	1627 (95.3)
Cannabis herb	1263	52 (4.1)	1211 (95.9)
Cocaine	676	42 (6.2)	634 (93.8)
Heroin	482	26 (5.4)	456 (94.6)
Other drugs	72	4 (5.6)	68 (94.4)
Total	4200	204 (4.9)	3996 (95.1)

## Discussion

The purpose of our study was to analyse all the seizures of suspected illicit substances confiscated by police forces from individuals who held them for personal use in two rich Italian provinces over 10 years, from 2008 to 2017. We found that seizures of cannabis, cocaine and heroin quantitatively prevailed in the investigated territory. However, from a qualitative point of view, especially from 2014 onwards, the range of substances seized, although always representing a smaller share (1.7%), was widening to include new psychoactive substances, especially dangerous stimulants such as synthetic cathinones and phenylethylamines. Overall, the trend of the seized illicit substances (Tables 1 and 2) was not linear. In 2012, a strong earthquake occurred in the two provinces, and this event could have interrupted the supply chain for infrastructure damage. However, in that year the number of seizures remained unchanged, while in the year after the number of seizures has significantly increased compared to all previous years (*P* < 0.05, Fisher’s exact test). After natural disasters, alcohol consumption, gambling and other psychiatric disorders have been reported to increase [23]. However, it is possible that the observed increase of seizures could also be attributed to greater control of the territory in the reconstruction phase.

In the two provinces studied, cannabis seizures (Fig. 4) from individuals between the ages of 14 and 24 reached almost 90%. Indeed, cannabis use, especially among adolescents, is increasing worldwide [24]. In our study, however, cannabis was the most seized substance in all age groups, in accordance with European data that indicate cannabis as the most widely used illicit substance by individuals aged 15 to 64 [14]. In the studied area, the seizures were predominately resin, but since the beginning of 2013 (Fig. 2, Table 1), seizures of cannabis herb, initially markedly lower than resin seizures, exceeded (or were slightly lower than) those of the resin. In the literature, the higher use of herbal cannabis compared to the one of resin cannabis has been related to the change in policy regarding the use and dissemination of herbal medical cannabis [25, 26]. In contrast to this interpretation, however, other authors reported that legalization for medical purposes is not associated with an increased frequency of cannabis use among adolescents [27, 28]. From the analyses carried out in this study, the median potencies (Δ^9^-THC content) of resin and herb were the same, approximately 9.5%, suggesting that these cannabis mixtures originated from the same cultivation sites and selected seeds for plants with high inflorescence production. In fact, in recent years, cannabis herb was mainly composed of inflorescence, whereas in the past, cannabis inflorescence was mixed with leaves [29, 30].

From 2008 to 2017, in the provinces of Modena and Reggio Emilia (Table 1), cocaine was the second most frequently seized substance after cannabis. The total seizures were 16.1%, greater than those in the Italian Florentine area in a similar time frame (10.47%) [31]; moreover, in 2016, significantly more seizures occurred in the study area (10.9%) than in Italy overall (6.6%), according to data available for the same year [32]. This high presence of cocaine in our provinces could be related to socio-economic factors. In particular, the wealth of the investigated territory could have favoured the use of cocaine, especially by adults with good economic status [33]. In agreement with this hypothesis, the average age of the subjects from whom cocaine was seized was high (32.6 ± 9.4 years), and cocaine (Fig. 4) was the most seized substance among subjects older than 55 years of age. The trend of seizures (Fig. 2) showed a decrease over the last 5 years; on the contrary, the purity of cocaine (Fig. 3) increased, with a median value reaching 75% in recent years. The same pattern has been reported at the European level [14, 32] and to a lesser extent in another Italian area [31].

In the studied provinces, the trend of heroin seizures (Fig. 2) sharply decreased over time. However, the hazards remained high because it showed very large purity variations (Fig. 3), increasing to a median value of 16.8% in 2017. Large variations in heroin purity have been associated with risk of overdose [34], as people using drugs do not know and do not perceive the purity of the substances they take [35]. Furthermore, in our investigation, heroin was the drug most often seized together with cocaine, which suggested that the injection of cocaine (“speedball”) by people using heroin [36] was common. This practice has negative consequences on health, social adaptation and treatment outcomes of opioid addiction [37].

The present study involved a specific Italian territory. However, the variety of seized substances reflects the dynamic nature of the global drug market. In conjunction with increasing reports of new psychoactive substances (NPS) in Europe [32, 38], seizure analysis (Table 2) indicated an expansion of the variety of psychoactive compounds available in the two provinces, to include novel substances commonly sold online [39]. These belonged to various pharmacological classes, but they mainly were synthetic stimulants (mephedrone, butylone, 4-MEC, 3-MMC, pentedrone, amphetamine, MDMA, 2C-B), followed by prescription opioids (methadone, buprenorphine, tramadol), hallucinogens (ketamine, LSD, psilocybe), prescription hypnotics (lormetazepam, zolpidem) and a synthetic cannabinoid agonist of the CB1 and CB2 receptors with high selectivity for CB2 receptors (UR-144) [40]. This range of psychoactive substances was wider than the one reported in the Italian Florentine area in a similar period [31]. Considering that synthetic cathinones and phenylethylamines have been associated with fatal intoxication [41–44], the situation was therefore alarming as these compounds are not detected by routine toxicological tests [15]. The diffusion of NPS found in our study was exclusively among young adults, in the age group 25–34 years.

Among the cases in which more than one substance was seized at the same time, there were two cases of tramadol, a prescription opioid with abuse potential [45], and tropicamide. This is an antimuscarinic drug used locally before eye examination for its cycloplegic and mydriatic effects. It appears to increase the efficacy of opioids and delay the onset of withdrawal symptoms [46].

In the European Union, males outnumber females among people using drugs [47]. Accordingly, in our study, most of the seizures (Table 3) involved male subjects without variations over the study period or in the type of substance and 82% of the seizures were from individuals younger than 35 years of age.

Our study has some limitations. The seizures reflected the operational priorities of the police forces. In fact, all the samples were seized by the police in accordance with Art. 75 D.P.R. 309/90 (possession for personal use). They were probably fewer in number than the total seizures made by the police forces in the provinces of Modena and Reggio Emilia. For this reason, the comparison with Italian and European statistics on seizures should be considered indicative as these statistics cover all types of seizures. The study was performed at the subnational level, however, our results have reported information about the illicit substances actually consumed in an Italian area, which is among the richest and industrialized in Europe. Moreover, the large number of samples analysed and the wide period considered, contributed to making our statistics significant in terms of trends of consumption and to showing changes in the drug market scenario. Finally, the developed analytical procedures were methodologically rigorous and consistently implemented to monitor the substances reported by the national early warning system (Italian National Institute of Health and Department for Anti-Drug Policies, SNAP project) [48].

In summary, our results indicated that old and new problems related to illicit substance use coexisted together in the investigated territory. When considering the older substances, the number of high purity cocaine seizures was worrying. In fact, the use of cocaine leads to a specific risk of vascular diseases and it is associated with increased emergency room visits [7]. Moreover, even if the number of heroin seizures was low, the risk of overdose for wide fluctuation in heroin purity persisted. At the same time, the spread of high potency herbal cannabis increased, especially among very young individuals. This phenomenon was alarming considering that cannabis use in adolescence has been associated with an increased risk of developing depression and suicidal behaviour later in life [24]. Finally, in recent years NPS have advanced, in particular the synthetic cathinones, which are associated with serious adverse reactions [49]. This emerging trend indicated that routine toxicological screening, largely unsuitable for NPS detection [50], should be replaced with specific and sensitive analytical methods to diagnose and treat, without delay, any intoxications from these novel substances.

## Conclusions

The persistence of old illicit drugs and the rapid emergence of new psychoactive substances represented a serious challenge for public health in the studied Italian area. We believe that analytical assessments carried out on seizures confiscated in accordance with Art. 75 D.P.R. 309/90 (possession for personal use) could provide a more detailed view of type and purity of the illicit substances intended for the final consumers. This information could be integrated with data from other monitoring systems to better identify the vulnerabilities of the territory and the priorities of intervention for both health agencies and contrast politics.

Finally, based on our results we suggest some examples of interventions, which could be useful: 1. information/education/prevention programs on the possible complications associated with the use of cannabis, aimed mainly at young people and involving the school, families and general practitioners; 2. standardized procedures to diagnose and effectively treat cocaine-related emergencies in hospitals; 3. increasing of naloxone distribution programs to antagonize possible heroin overdoses, and providing to those known to the local drug dependence units information on the great variability of purity of the heroin that circulated in the territory and on the risks of overdose that this entails; 4. equipping the laboratories to carry out the analytical chemical identification of the NPS, both to allow clinicians to recognize and treat any acute intoxications reaching emergency departments and to report to the national early warning system.

## Supplementary information

**Additional file 1.** Chemicals and reagents; Sample processing for untargeted analyses; Sample processing for quantitative targeted analyses; Working solutions and calibration curves; GC-FID method; GC-MS method; LC-MS/MS method; Quantitative analysis and method validation.

## Data Availability

The datasets used and analysed during the current study are available from Dr. M. Licata on a reasonable request.

## Abbreviations

2C-B: 4-bromo-2,5-dimethoxybenzeneethanamine
3-MMC: 3-methylmethcathinone
4-MEC: 4-methylethcathinone
D.P.R: Decree of the president of the Italian Republic
GC-FID: Gas chromatography-flame ionization detector
GC-MS: Gas-chromatography-mass spectrometry
LC-MS/MS: Liquid chromatography-tandem mass spectrometry
LSD: D-lysergic acid diethylamide
MDMA: 3,4-methylene-dioxymethamphetamine
NPS: New psychoactive substance
SD: Standard deviation
Δ^9^-THC: Δ^9^-tetrahydrocannabinol
