# Supplementary material for: Seizures of illicit substances for personal use in two Italian provinces: analysis of trends by type and purity from 2008 to 2017
Source: Subst Abuse Treat Prev Policy. 2019 Sep 18;14:41. doi: 10.1186/s13011-019-0229-y (PMC6751801; doi:10.1186/s13011-019-0229-y)
Supplement: Supplementary file 1 — Additional file 1. Chemicals and reagents; Sample processing for untargeted analyses; Sample processing for quantitative targeted analyses; Working solutions and calibration curves; GC-FID method; GC-MS method; LC-MS/MS method; Quantitative analysis and method validation. [file 13011_2019_229_MOESM1_ESM.docx]

**Additional file 1**

**Chemicals and reagents**

Certified reference standards of the target analytes (as free bases or salts) were supplied as pure substance or solution by Merck-Sigma Aldrich (St. Louis, MO, USA) or by LGC (LGC Standards S.r.l., Milano, Italy). Docosane and (-)-scopolamine hydrochloride were used as internal standards (ISs) and were purchased as powders from Sigma (St. Louis, MO, USA).

All solvents and chemicals used during laboratory work were of analytical reagent grade. Ultrapure water (Merck Millipore Direct-Q8, Germany) with a resistivity of 18 MΩ.cm was used to prepare solutions during the experimental process. All solvents for LC-MS/MS were of LC-MS purity grade (Baker-VWR, Milano, Italy).

**Sample processing for untargeted analyses**

A representative sampling procedure was performed to yield samples truly representative of the whole seizure, and solid materials were ground into fine particles before processing. Test samples (20 mg) were added with an adequate volume of methanol and sonicated for 10 min. The obtained supernatants were directly subjected to GC-MS or LC-MS/MS for the analysis of unknown (untargeted) compounds.

**Sample processing for quantitative targeted analyses**

For cannabis samples (herb or resin), 150 mg aliquots were added with 10 mL of petroleum ether (40-60°) containing docosane as an IS and sonicated for 10 min at room temperature. The extraction procedure was repeated two times, and the obtained clear supernatants were combined prior to analysis; the nominal IS concentration was 1.0 mg/mL. For heroin and cocaine, 20 mg powder samples were added with 10 mL of methanol containing scopolamine as an IS and vortexed, and the obtained clear extracts were subjected to analysis. The nominal IS concentration was 1.0 mg/mL. The obtained supernatants were directly subjected to GC-FID analyses for quantification of the target illicit substances.

**Working solutions and calibration curves**

Working solutions (n=10) of Δ^9^-tetrahydrocannabinol (Δ^9^-THC), cocaine and heroin were prepared in the range 0.1-2.0 mg/mL for heroin and cocaine and 0.1-7.0 mg/mL for Δ^9^-THC; the concentration of the IS in the calibrators was 1.0 mg/mL for both docosane and scopolamine. All solutions were stored at -20°C until use. The calibrators were subjected to GC-FID analyses in triplicate on separate days, and the regression curves were calculated from the peak area ratios of the analyte to the internal standard versus the nominal analyte concentration by a weighted (1/x) linear regression model.

**GC-FID method**

GC-FID analyses were performed using a CP-3880 gas chromatograph (Varian Inc., Palo Alto, CA, USA) equipped with a CombiPAL GC sample injection system (CTC Analytics, Agilent Technologies, Palo Alto, CA) and a flame ionization detector (FID) (Varian Inc., Palo Alto, CA, USA). Chromatographic separation was achieved on a DB-5MS fused silica capillary column (30 m × 0.25 mm i.d.; film thickness, 0.25 μm) (Agilent Technologies, Palo Alto, CA). The oven temperature was programmed at 100°C (5.0 min), and the temperature increased at the rate of 10°C/min to 290°C, which was maintained for 5.0 min. The injection volume was 1.0 μL with a split ratio of 30:1. The injector temperature was maintained at 200°C, and the detector temperature was set at 300°C. Ultrapure helium was used as the carrier gas at a flow rate of 1.0 mL/min.

**GC-MS method**

Analyses were performed with a CP-3800 gas chromatograph equipped with a CP-8400 autosampler and coupled to a Saturn 2200 ion trap mass spectrometer operating under electron impact ionization (EI) mode. Data acquisition and analyses were performed using MS Workstation 6.3 software (Varian Inc., Palo Alto, CA, USA). Analyte separation was achieved on a fused silica capillary column (DB-5MS; 30 m × 0.25 mm i.d.; film thickness, 0.25 μm) (Agilent Technologies, Palo Alto, CA). The oven temperature was programmed to maintain 100°C for 5 min, increase to 300°C at 10°C/min and finally maintain 300°C for 13 min. The carrier gas was helium (purity 99%) with a flow rate of 1.0 mL/min. The splitless injection volume was 1.0 μL. The injection port, ion source, ion trap, and interface temperatures were 200°C, 150°C, 150°C and 250°C, respectively. MS acquisition was performed in full scan mode in the *m/z* range of 43-510. Qualitative analyses were performed by comparing the retention time and mass spectrum of each analyte to those of the certified standards analysed under the same experimental conditions. Illicit substances were also identified by matching experimental full scan spectra against the NIST spectral library, the most powerful database for unknown screening.

**LC-MS/MS method**

LC-MS/MS analyses were performed on an Agilent 1200 LC system consisting of a binary pump, an autosampler, an on-line degasser and a thermostatted column compartment (Agilent, Waldbronn, Germany). Chromatographic separation was achieved on a Synergi^TM^ Polar-RP 80 A column (150 × 2 mm; 4 μm particle size) preceded by a Polar-RP Security Guard Cartridge (4 × 2 mm) (Phenomenex, Bologna, Italy); the column temperature was set to 40°C. Elution was performed with a mobile phase of (A) 1.0 mM aqueous ammonium acetate (+0.1% formic acid) and (B) 0.1% formic acid in acetonitrile/methanol (70/30; v/v) with a flow rate of 0.25 mL/min and using the following gradient: 0.0-20.0 min, linear gradient from 5 to 95% (B); 20.0-23.0 min, isocratic at 95% (B); and 23.0-23.3 min, linear gradient from 95 to 5% (B). The pre-equilibration period between runs was 6.7 min. The injection volume was 10 μL, and the autosampler was maintained at room temperature.

Tandem mass spectrometric detection under multiple reaction monitoring mode (MRM) was accomplished by a SCIEX API4000 QTRAP mass analyser equipped with a Turbo Ion Spray source (SCIEX Toronto, Canada) operating both in the positive and negative ESI modes. A dwell-time of 20 ms was used for all transitions. The following optimized instrument settings were used: curtain gas, 10 psi; CAD gas, 4 psi; nebulizer gas (GS1), 35 psi; heater gas (GS2), 45 psi; ion spray voltage, 4000 V; and temperature, 450°C. The nitrogen flow was produced by a nitrogen generator model 75–72 (Whatman Inc., MA, USA). The mass spectrometer was calibrated to <2.0 mDa mass error prior to each batch analysis. The Analyst Software (version 1.5.1) was used for instrument control, data acquisition, and data elaboration.

MRM transition values for illicit drug identification were obtained from the literature [17–20] and confirmed for each analyte together with the corresponding retention time by LC-MS/MS analysis of reference standard for unambiguous identification.

**Quantitative analysis and method validation**

Samples found to be positive for Δ^9^-THC, cocaine and heroin, i.e., the most prevalent illicit substances, were subjected to sample processing for targeted analyses and to quantitative analysis by GC-FID. The optimized GC-FID method was fully validated according to the Scientific Working Group of Forensic Toxicology (SWGTOX) standard practices for method validation in forensic toxicology [21] and according to the GTFI (Group of Italian Forensic Toxicologists) guidelines [22]. All calibration curves exhibited R^2^ values > 0.990, and the validation parameters fulfilled the acceptance criteria.
